# Seasonality and relative abundance within an elasmobranch assemblage near a major biogeographic divide

**DOI:** 10.1371/journal.pone.0300697

**Published:** 2024-06-26

**Authors:** Grace Roskar, James W. Morley, Jeffrey A. Buckel

**Affiliations:** 1 Institute of Marine Sciences, University of North Carolina at Chapel Hill, Morehead City, North Carolina, United States of America; 2 Department of Biology, Coastal Studies Institute, East Carolina University, Wanchese, North Carolina, United States of America; 3 Department of Applied Ecology, Center for Marine Sciences and Technology, North Carolina State University, Morehead City, North Carolina, United States of America; Japan Fisheries Research and Education Agency, JAPAN

## Abstract

Nearshore waters are utilized by elasmobranchs in various ways, including foraging, reproduction, and migration. Multiple elasmobranch species have been previously documented in the nearshore waters of North Carolina, USA, which has a biogeographic break at Cape Hatteras on the Atlantic coast. However, comprehensive understanding of the elasmobranch community in this region is still lacking. Monthly year-round trawling conducted along two ocean transects (near Cape Lookout and Masonboro Inlet in 5 to 18 m depth) in Onslow Bay, North Carolina provided the opportunity to examine the dynamics and seasonal patterns of this community using a multivariate approach, including permutational multivariate analysis of variance and nonparametric BIO-ENV analysis. From November 2004 to April 2008, 21,149 elasmobranchs comprised of 20 species were caught, dominated by spiny dogfish (*Squalus acanthias*) and clearnose skate (*Rostroraja eglanteria*). All species exhibited seasonal variation in abundance, but several key species contributed the most to seasonal differences in species composition within each transect. Spiny dogfish was most abundant in the winter at both locations, comprised mainly of mature females. Although clearnose skate was caught in all seasons, the species was most abundant during the spring and fall. Atlantic sharpnose (*Rhizoprionodon terraenovae*) was one of the most abundant species in the summer, and two distinct size cohorts were documented. Temperature appeared to be the main abiotic factor driving the community assemblage. The extensive year-round sampling provided the ability to better understand the dramatic seasonal variation in species composition and provides new information on the relative abundance of several understudied elasmobranch species that may be of significant ecological importance. Our results underscore the importance of inner continental shelf waters as important elasmobranch habitat and provide baseline data to examine for future shifts in timing and community structure at the northern portion of the biogeographic break at Cape Hatteras.

## Introduction

Nearshore waters, including coastal waters up to 20 m in depth, represent productive and dynamic environments that contain species rich communities [1,2]. Elasmobranchs (i.e., sharks, skates, and rays) are one group of many taxa that inhabit these waters, and they comprise the majority of larger-bodied fauna [3]. Elasmobranchs utilize nearshore habitats for foraging and reproduction [1], as nursery areas [4–6], and as migratory pathways [7]. While nearshore waters represent critical habitat for many species of elasmobranchs, these areas also have a relatively high exposure to anthropogenic impacts. These manmade threats include commercial and recreational fishing, where elasmobranchs may either be targeted or caught as bycatch, and habitat degradation resulting from pollution and coastal development activities such as dredging of navigation channels [1,8–10]. An understanding of the seasonal community dynamics for this assemblage is an important step towards ecosystem-based management within this system that serves a variety of natural and human-use functions. As marine policy shifts towards implementing ecosystem-based approaches [11], it is increasingly important to elucidate habitat use and seasonal distributions of species [12–14].

As predators and prey in coastal systems [15], sharks and rays can have various roles in food web dynamics, from trophic cascades to resource partitioning to ecosystem engineering through bioturbation [16–20]. A better understanding of elasmobranch assemblages is also important for conservation reasons, because a quarter of chondrichthyan species that inhabit coastal and continental shelf waters are listed as threatened by the IUCN Red List [10]. It is increasingly important to understand what drives the structure of elasmobranch communities amidst population declines due to overfishing and habitat loss for some species [10,21], while increases in population size occur for others [22]. Understanding and preventing elasmobranch declines is made more difficult due to poorly refined fisheries landings categories [23] and a lack of adequate management actions to prevent depletion [24]. Domestic management measures have been undertaken to combat population declines, such as the first federal Fishery Management Plan (FMP) for sharks implemented in 1993 [25] and later the mandate for FMPs to identify essential fish habitat (EFH) for every life stage of managed species [26]. However, estimating abundance of many elasmobranch species remains a challenge [27], in part due to a lack of understanding on seasonal distributions and habitat use [28–30].

North Carolina occupies a unique position along the United States Atlantic coast. In particular, Cape Hatteras lies at the confluence of temperate waters originating from the Labrador Current and warmer waters originating from the Gulf Stream, which creates a thermal and hydrographic barrier [31]. As a result, this region represents one of the most significant biogeographic breaks in the world [32,33]. The coastal waters of North Carolina contain a seasonally variable assemblage of both temperate and tropical species [34,35] and also represents an important migratory corridor for seasonal movements of fish and elasmobranchs [36,37]. Historically, over 50 species of elasmobranchs have been recorded in the nearshore waters of North Carolina [38].

While shark communities in several coastal and estuarine areas of the southeast U.S. have been examined [6,39–42], significant gaps remain. For example, to our knowledge no studies in nearshore coastal waters have consistently sampled throughout the entire year. Most studies have focused only on sharks, despite the fact that batoid species have been shown to comprise a significant portion of the elasmobranch biomass in continental shelf ecosystems [3,43–45]. One notable exception to the lack of data on batoid species is the Southeast Area Monitoring and Assessment Program South Atlantic trawl survey (SEAMAP-SA), which has operated since 1989 during spring, summer, and fall. In this survey batoid species are counted, but not measured, and sampling is not conducted during the winter [3]. The present study represents one of the first analyses of the entire elasmobranch community in the nearshore waters of North Carolina throughout the entire year. We utilize data from a trawl survey conducted between 2004 and 2008, which was originally purposed to examine bluefish (*Pomatomus saltatrix*) recruitment [36], but also recorded data on over 20,000 individual sharks, rays and skates in North Carolina. The aims of this study were to characterize the elasmobranch assemblage caught in two different areas of the North Carolina coast and to identify spatial or seasonal patterns in species distributions and relative abundances to determine what environmental factors drive community responses.

## Materials and methods

### Elasmobranch sampling

**Fig 1:**
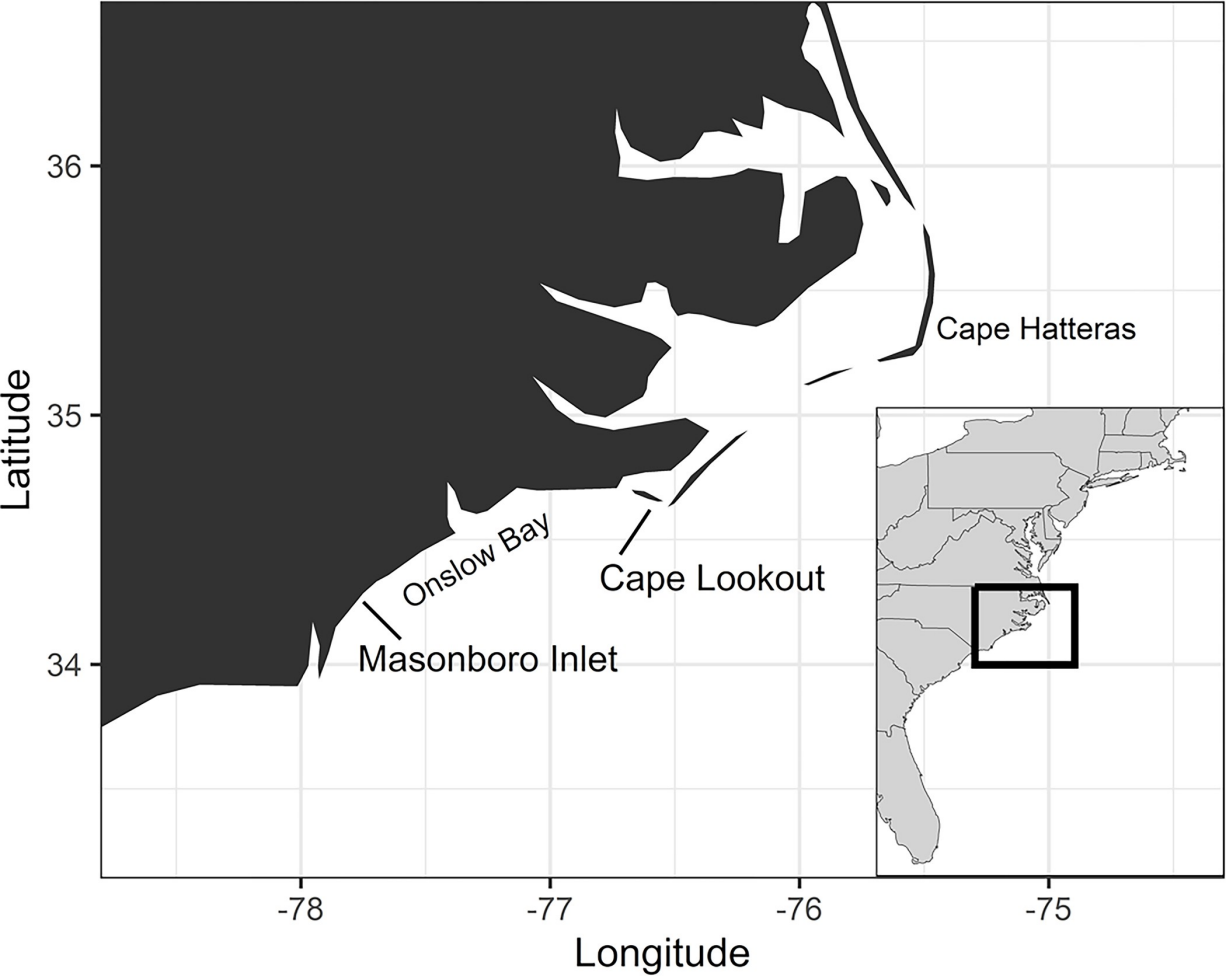
Study area showing Cape Lookout and Masonboro Inlet transects. The map was made using R packages *ggplot2* and *maps* packages in R [46,47].

Monthly trawling was conducted in Onslow Bay, North Carolina from November 2004 to April 2008 [36]. The sampling was originally planned to assess bluefish (*Pomatomus saltatrix*) recruitment patterns across the U.S. Atlantic coast [36]. Further, there were limited funds for year-round sampling, so for certain years more targeted effort took place to sample key months for bluefish off North Carolina. Thus, there was not continuous monthly effort during the study period, which began in the fall of 2004 (Table 1). Two transects were sampled, which were oriented perpendicular to shore: one extending southward from Shackleford Banks to the west of Cape Lookout (76°36’W, 34°39’ N; referred to hereafter as Cape Lookout) and the other extending eastward off Masonboro Inlet (77°47’W, 34°13’N) (Fig 1). These two transects differed in several ways. The Cape Lookout transect was geographically closer to the biogeographic break at Cape Hatteras, it was in close proximity to a prominent cape, and the adjacent estuarine system was more extensive. By contrast, the Masonboro Inlet transect was in a region where hardbottom habitat is more common, although this type of bottom was avoided by the trawl.

**Table 1:** Number of tows conducted at each transect (CL = Cape Lookout, MI = Masonboro Inlet) per month and season from 2004–2008.

Season	Month	2004		2005		2006		2007		2008		Total # tows	Tows per season
		CL	MI	CL	MI	CL	MI	CL	MI	CL	MI		
Winter	January	-	-	5	6	6	6	-	-	6	6	35	69
	February	-	-	6	4	-	-	-	-	-	-	10	
	March	-	-	6	6	6	6	-	-	-	-	24	
Spring	April	-	-	6	6	6	-	-	-	6	-	24	59
	May	-	-	6	6	5	6	-	-	-	-	23	
	June	-	-	6	6	-	-	-	-	-	-	12	
Summer	July	-	-	6	6	6	6	-	-	-	-	24	71
	August	-	-	6	6	6	6	-	-	-	-	24	
	September	-	-	6	6	5	6	-	-	-	-	23	
Fall	October	-	-	-	-	-	-	-	-	-	-	0	93
	November	6	3	6	6	6	6	6	6	-	-	45	
	December	6	6	6	6	6	6	6	6	-	-	48	

Each transect consisted of six stations–0.4, 0.8, 1.6, 3.2, 5.6, and 8 km from shore. The four stations farther inshore were in the 5–9 m depth range while the two farther offshore stations were in the 10–18 m depth range. Sampling was generally conducted at each transect once per month using a three-in-one bottom otter trawl net that was modeled after a trawl used by the New Jersey Department of Environmental Protection for their annual Ocean Stock Assessment Survey of coastal resources [48]. The trawl had a 25 m head rope and 30.5 m footrope. The stretched mesh size was 12 cm in forward netting and 8 cm in the codend, and a 1 cm liner was installed in the codend. Steel trawl doors (1.8 m length, 324 kg) were separated from the net by 55 m of rubber-reinforced steel cable (including the bridle and ground cable). The sampling was conducted onboard the F/V Capt. David, a 21 m long wooden shrimp trawler equipped with outriggers and a net reel on the stern. Each trawl door was connected via steel cable to the separate outriggers on the vessel, to maximize net spread. For each trawl tow, the net was pulled parallel to shore at 3 knots for 20 minutes.

For our analysis we assumed that all hauls were equivalent and represented a unit of effort, which was necessary as geographic coordinates were not consistently recorded at the start and end of each haul, which prevented a more meaningful calculation of area swept. Sampling effort varied over the course of the study, with the most consistent effort occurring between November 2004 and December 2006 (Table 1). Seasons were defined as winter = January-March; spring = April-June; summer = July-September; fall = October-December (Table 1). A handheld YSI-brand water quality meter, equipped with temperature, salinity and dissolved oxygen sensors was used to collect water quality data near the surface and bottom (up to 15 m cable depth). Temperature (⁰C), salinity (ppt), and dissolved oxygen (DO, mg/L) were measured near the surface (< 0.3 m) and approximately 1 meter off the bottom for each trawl tow. At the most offshore station, the depth was typically greater than the length of the YSI cable, so the water quality measurements were taken at the maximum cable length of 15 m, which was estimated to be within 4 m of the bottom.

All elasmobranchs were identified, sexed, and counted for each tow. Southern stingrays (*Hypanus americanus*) and bluntnose stingrays (*Hypanus say*) were aggregated to the genus level (*Hypanus spp*.) due to the possibility of misidentification of smaller individuals early in the survey. The fork length (FL) of sharks and disc width (DW) of skates and rays was measured to the nearest mm. If more than 30 individuals of a species were caught in a single tow, all individuals were counted but only a subsample of 30 random individuals was measured. This research was conducted under Protocols 02-066-O and 05-089-O, approved by North Carolina State University’s Institutional Animal Care and Use Committee (IACUC).

### Data analyses

The number of individuals were summed for each transect and each season per taxon/taxa. A chi-square goodness of fit test [49] was used to test for differences between observed and expected (1:1) sex ratios for species that comprised ≥1% of the total elasmobranch catch in any season (*n* = 10). For these same ten species we used the Kolmogorov-Smirnov test to examine differences in length distributions between sexes. A length-frequency histogram was constructed and the mean ± standard deviation for each sex was calculated for the ten species. We compared observed lengths with sex-specific estimates of length at sexual maturity, which were obtained from available published literature [50–62]. Individuals in this study were not dissected for examination of reproductive organs to assess maturity; therefore maturity was assumed based on published estimates of lengths at sexual maturity. If estimated lengths at sexual maturity were not published as fork length, published equations were used to convert total length to fork length [51,58]. The lengths at sexual maturity for the *Hypanus spp*. group were not assessed due to the presence of two species (southern and bluntnose stingrays) with different lengths at sexual maturity.

A two-factor permutational multivariate analysis of variance (PERMANOVA) [54] tested the null hypotheses that there were no significant differences in species composition (of the ten species that comprised ≥1% of the total elasmobranch catch in any season) between transects (Cape Lookout versus Masonboro Inlet) or among seasons. The response variable for the PERMANOVA was catch-per-unit effort (CPUE), calculated as the number of individuals caught per trawl tow. Catch-per-unit-effort for positive tows (tows that caught at least one elasmobranch, *n =* 290) were square-root transformed before the Bray-Curtis similarity matrix was constructed, to reduce the influence of highly abundant species compared to less abundant species [63]. Type III (partial) sums of squares were used due to the unbalanced data, and 999 unrestricted permutations of the CPUE data were used due to the two-factor crossed design of the PERMANOVA [63]. Upon a significant PERMANOVA result (*p* ≤ 0.05), the similarity percentage (SIMPER) analysis was used on the square-root transformed data to identify which species contributed to the differences in species composition between transects or among seasons [64]. All PERMANOVA and SIMPER analyses were run using Primer software v.7.0.13 [65].

Non-metric multidimensional scaling (nMDS) ordinations were used to visualize seasonal and spatial differences in the elasmobranch community. Ordinations were created for each season and for each transect, as well as an ordination for the entire elasmobranch community sampled. Samples (i.e., each trawl tow) were arranged in a two-dimensional space with the relative distance between samples indicating the pairwise similarities in rank order [66]. The nMDS ordinations were created using a Bray-Curtis similarity matrix of square-root transformed CPUE data. Stress, or the fit between similarity rankings and the corresponding distance rankings in the ordination space [66], was calculated for each nMDS ordination to determine the level of preservation of Bray-Curtis sample dissimilarities by Euclidean distances [67,68]. Stress levels < 0.2 are generally considered acceptable [69].

The nonparametric BIO-ENV analysis was used to examine the relationships between the elasmobranch community and environmental variables among both transects pooled as well as separately. The BIO-ENV analysis determines the subset of environmental variables that have the greatest Spearman rank correlation (rho, ρ) with sample dissimilarities [70]. A Euclidean distance-based resemblance matrix was constructed with the standardized environmental data (temperature, salinity, distance from shore) and a Bray-Curtis similarity matrix was constructed with the square-root transformed community (CPUE) data. If trawl tows were missing either bottom or surface measurements of any of the environmental variables measured (temperature, salinity), linear interpolation was used to estimate missing values using trawl hauls from adjacent locations on the same day. The means of the bottom and surface measurements for each trawl tow were used in the analysis, as no strong stratification was evident between the surface and bottom temperature data. Surface and near-bottom temperatures (i.e., within 1 m of surface and bottom) were highly correlated (slope = 0.93, r^2^ = 0.99, N = 101). On average, surface temperatures were 0.41 C warmer than bottom temperatures (median = 0.10 warmer at surface), and only 6 observations had surface temperatures greater than 1.5 C warmer at the surface (max 2.9 C). The BIO-ENV analysis was also performed on the species-level biological data to determine the group of species most related to (i.e., the greatest rank correlation with sample dissimilarities) the differences in seasonal communities for each transect, as well as for the overall community sampled in Onslow Bay (i.e., across both transects).

The results of the BIO-ENV analyses were plotted on the nMDS ordinations. Each environmental variable and each species in the most parsimonious BIO-ENV models were plotted as vectors on separate plots of the nMDS ordination. Each species in the best fitting BIO-ENV model for individual seasons were also plotted as vectors on nMDS ordinations for the given season. The *metaMDS*, *bioenv*, and *envfit* functions in the R package *vegan* were used to perform the nMDS ordinations, BIO-ENV analyses, and fitting of environmental variables and species, respectively [71].

## Results

Overall, 21,149 elasmobranchs of 20 species were caught in 290 of 292 trawl tows between November 2004 and April 2008. The ten shark species caught comprised 55% (*n* = 11,558) of the total elasmobranch catch in numbers, while the ten batoid species caught comprised 45% (*n* = 9,591; Table 2). All 20 species were caught along the Cape Lookout Transect while species richness was lower at the Masonboro Inlet transect (*n* = 15). The ten elasmobranch species that individually comprised ≥ 1% of the elasmobranch catch in any season made up 99% (*n* = 21,052) of the combined elasmobranch catch. Spiny dogfish (*Squalus acanthias*) was the most abundant species overall (*n* = 6,270), comprising 30% of the total elasmobranch catch, and females were much more common than males (Table 2). Female Atlantic stingrays (*Hypanus sabinus*) were also caught in significantly higher numbers than males. By contrast, significantly greater proportions of male clearnose skates (*Rostroraja eglanteria)*, *Hypanus spp*., and smooth butterfly rays (*Gymnura lessae*) were caught compared to females (Table 2).

**Table 2:** Summary of elasmobranchs caught during 2004–2008 trawl surveys in order of abundance. The chi-square test results comparing differences between observed and expected (1:1) sex ratios for the species that comprised ≥1% of total elasmobranch catch in any season are reported with χ^2^ and *p*-values.

Species	n	Transect		Season				Temperature (°C)		Sex		*x*^2^ *(p)*
		Cape Lookout	Masonboro Inlet	Winter	Spring	Summer	Fall	Mean	Range	Female	Male	
Spiny dogfish *Squalus acanthias*	6270	3666	2604	5058	7		1205	12.46	9.25–15.60	1750	235	1156.3 (< 0.001)
Clearnose skate *Rostroraja eglanteria*	5012	2895	2117	1194	1983	158	1677	16.59	9.25–28.60	1221	2226	293.02 (< 0.001)
Smooth dogfish *Mustelus canis*	4376	3126	1250	940	2509	11	916	15.66	10.80–27.50	730	704	0.47141 (0.4923)
Smooth butterfly ray *Gymnura micrura*	2114	1594	520	75	924	689	426	19.80	12.00–29.10	606	759	17.149 (< 0.001)
Bullnose ray *Myliobatis freminvillii*	1007	432	575		510	15	482	20.29	16.80–29.05	398	355	2.4555 (0.1171)
Atlantic sharpnose shark *Rhizoprionodon terraenovae*	828	395	433		133	660	35	23.83	15.15–29.30	303	343	2.4768 (0.1155)
Hypanus spp. *Hypanus spp*.	764	514	250	2	355	266	141	21.49	13.70–29.30	288	351	6.2113 (0.01269)
Atlantic stingray *Hypanus sabinus*	609	203	406	199	3	57	350	16.27	10.80–28.20	286	235	4.9923 (0.02546)
Bonnethead *Sphyrna tiburo*	48	12	36		10	38		24.13	16.95–28.90	25	21	0.34783 (0.5553)
Cownose ray *Rhinoptera bonasus*	36	25	11	1	27	2	6	17.47	13.10–20.50	22	6	
Spiny butterfly ray *Gymnura altavela*	32	30	2		6		26	19.71	15.10–21.45	16	7	
Blacknose shark *Carcharhinus acronotus*	24	8	16		3	21		26.30	22.60–28.00	15	9	1.50 (0.2207)
Winter skate *Leucoraja ocellata*	10	8	2	9	1			13.00	12.00–16.05	6	3	
Roughtail stingray *Dasyatis centroura*	5	2	3		2	2	1	22.62	16.90–27.25	1	4	
Sandbar shark *Carcharhinus plumbeus*	4	4			4			15.18	15.15–15.20	2	2	
Sand tiger *Carcharhinus taurus*	3	3			2		1	17.50	15.45–20.10	1	2	
Scalloped hammerhead *Sphyrna lewini*	3	2	1		1	2		25.10	20.90–27.60	2	1	
Atlantic torpedo *Tetronarce occidentalis*	2	2			2			16.08	15.20–16.95	-	1	
Atlantic angel shark *Squatina dumeril*	1	1			1			18.60	18.60–18.60	-	-	
Common thresher shark *Alopias vulpinus*	1	1			1			16.80	16.80–16.80	-	-	

**Fig 2:**
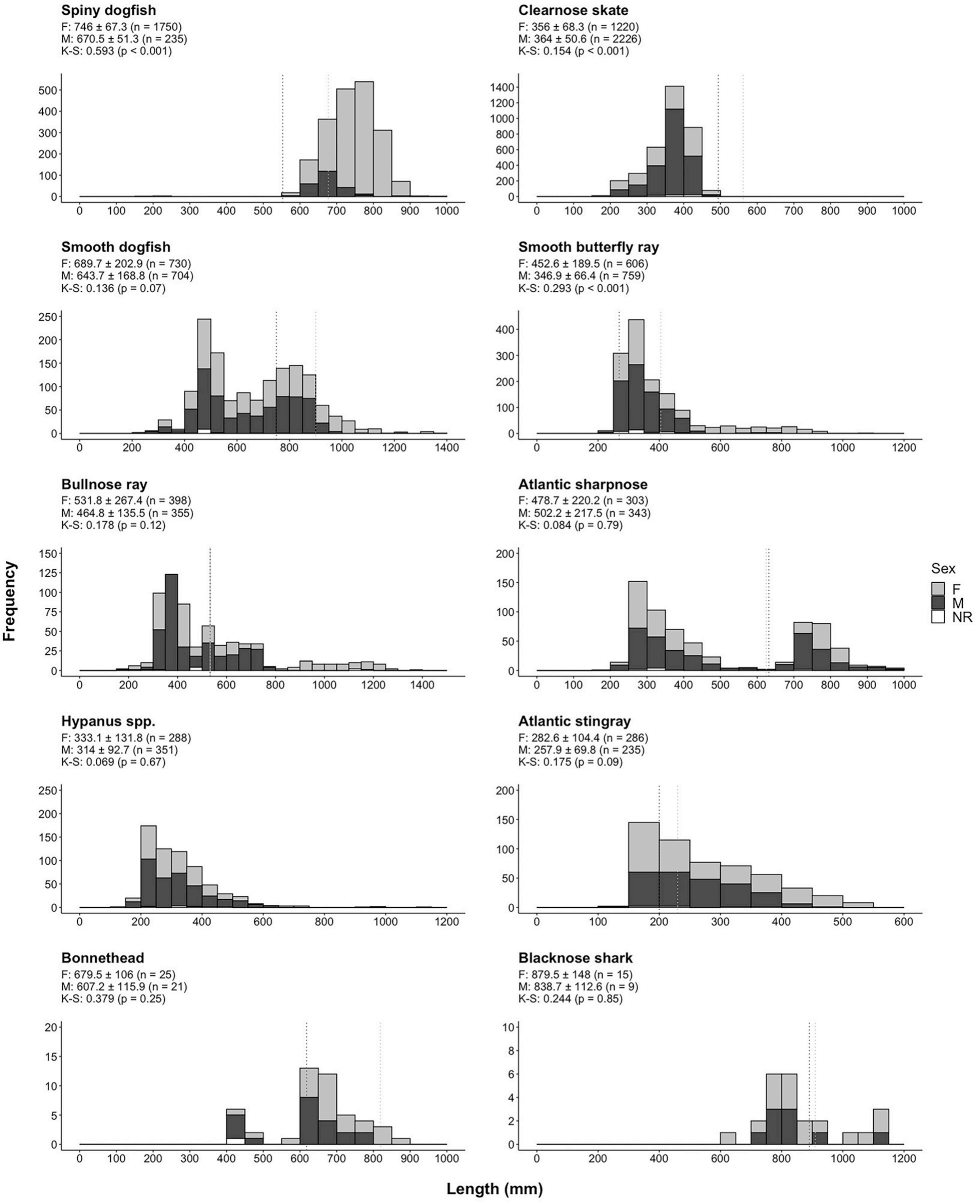
Length frequency histograms for species that comprised ≥1% of total elasmobranch catch in any season. Mean ± SD lengths are shown for females and males of each species. The results of the Kolmogorov-Smirnov (K-S) test for comparing differences in species-specific length distributions are reported underneath the mean lengths, with *p*-values in parentheses. The vertical dotted lines represent lengths at sexual maturity for each sex (dark gray dotted lines = male; light gray dotted lines = female). NR = not recorded.

A number of species exhibited differences in lengths between the sexes and most species consisted of both mature and immature individuals, which is assumed in our study based on published lengths at sexual maturity (Fig 2). The Kolmogorov-Smirnov test showed significant differences in the length distributions between males and females for spiny dogfish (Fig 2). Most spiny dogfish were greater than 600 mm FL and consisted of a significantly greater proportion of females. The majority of both female spiny dogfish (n = 1,502; 86%) and male spiny dogfish (*n* = 232; 99%) were mature. The opposite pattern was observed in smooth dogfish, where mature females (*n* = 117; 16%) and males (*n* = 247; 35%) made up a smaller percentage of individuals. Clearnose skate lengths showed a clear cohort of immature individuals, and only 3 mature individuals were caught (male). The K-S tests showed a significant difference in the length distributions between sexes of the clearnose skate; individuals between 200–300 mm DW consisted of significantly more females whereas more males comprised the 300–450 mm DW size range. Clearnose skates over 450 mm DW were mostly female, although the number of individuals greater than 450 mm DW was low (*n* = 42). The K-S test also indicated a significant difference in length distribution between sexes of the smooth butterfly ray; the majority (95%; n = 182) of smooth butterfly rays greater than 450 mm DW were female. However, less than half (44%) of the female smooth butterfly rays were mature (*n* = 266) whereas the proportion of males larger than the size at sexual maturity was much greater (96%; *n* = 725). A similar trend was observed in Atlantic stingrays, with about half of the female Atlantic stingrays caught as mature (53%; *n* = 153) whereas the majority of males were mature (74%; *n* = 173). As for bullnose rays (*Myliobatis freminvillii*), nearly equal percentages of both sexes of bullnose rays were mature: 31% of females (*n* = 122) and 30% of males (*n* = 107).

The shark species generally showed evidence of distinct cohorts based on length, with the exception of spiny dogfish, which did not have distinct size groups. For Atlantic sharpnose, the two cohorts represented a neat divide between smaller-immature individuals, and a larger-mature cohort (Fig 2) that contained 33% of females and 40% of males. Conversely, the batoid species tended to have more continuous length distributions, that were either skewed towards smaller (e.g., clearnose skate) or larger (e.g., Atlantic stingray) individuals (Fig 2).

**Fig 3:**
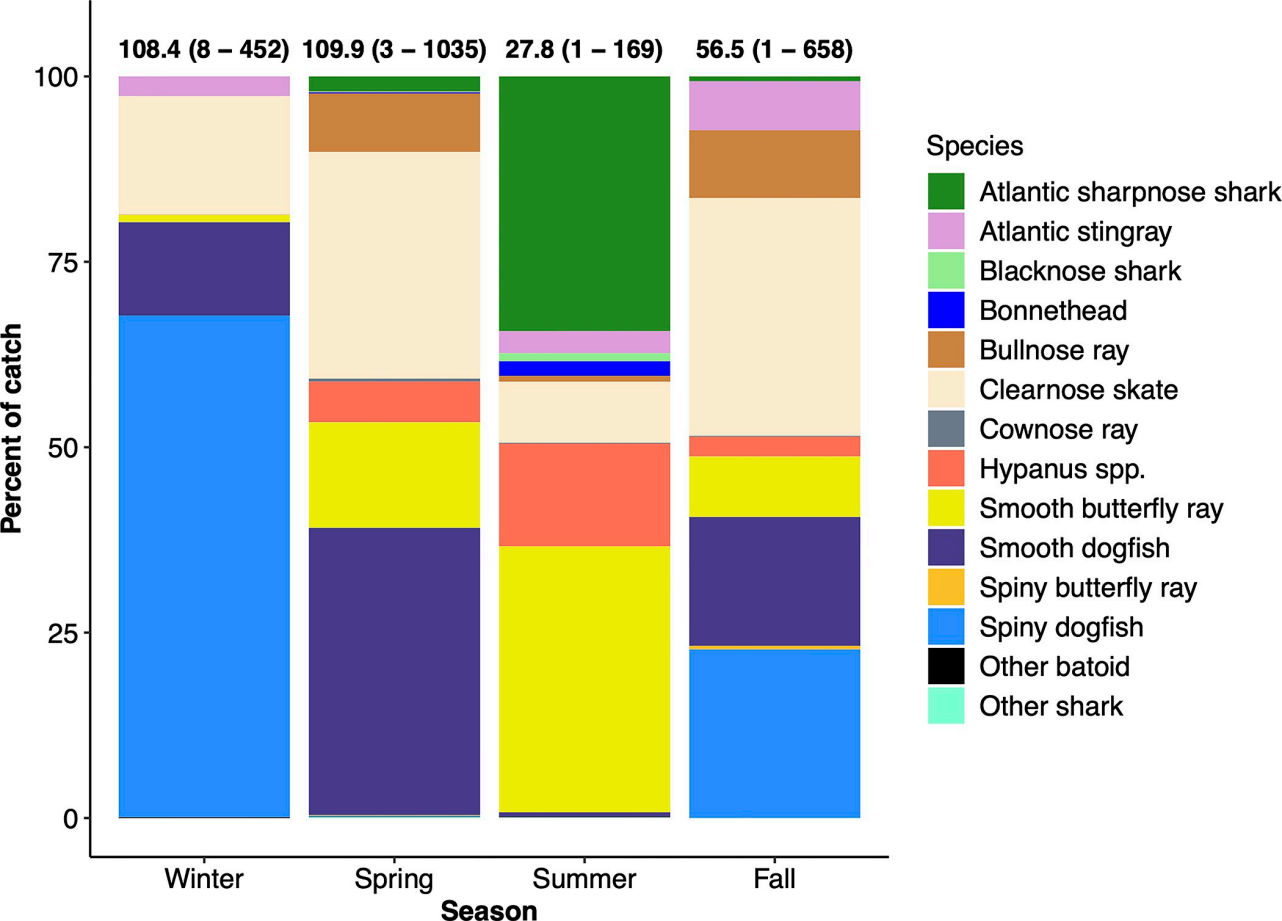
Seasonal composition of the elasmobranch catch. Numbers over bars represent the mean (range) number of individuals caught per tow for each season.

**Fig 4:**
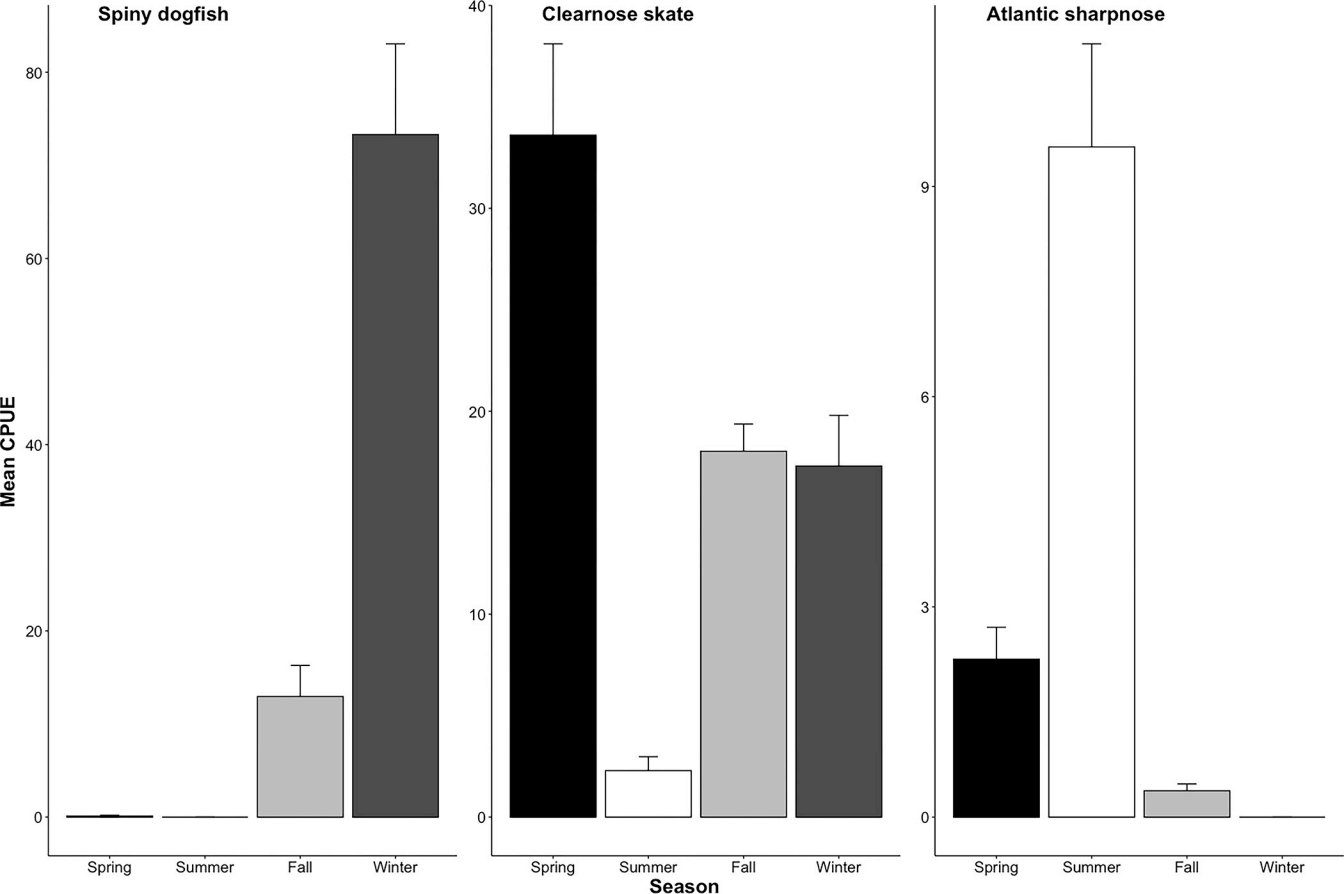
Mean (± SD) seasonal CPUE (individuals caught per tow) for three commonly caught species.

Elasmobranchs were caught across a wide temperature range of 9.3–29.3°C (mean ± SD = 17.8 ± 5.7°C) and a salinity range of 30.0–37.2 ppt (34.2 ± 1.34 ppt). While effort varied in the number of tows per season (Table 1), the mean number of individuals caught in a tow was greatest in the spring (mean = 109.9), and the lowest mean number of individuals caught per tow occurred in the summer (mean = 27.8; Fig 3). The winter catch, while lowest in species richness (*n =* 8) out of all seasons, was dominated by spiny dogfish, which comprised 68% of the season’s total elasmobranch catch (Figs 3 and 4). The spring season showed the greatest species richness, with all 20 species represented (Table 2). The PERMANOVA analysis showed a significant difference in species composition between transects and among seasons and these two factors also had a significant interactive effect (Table 3). Subsequent pairwise PERMANOVA analysis showed that the species composition of each season was significantly different (*p* = 0.001) from all other seasons (S2 Table). Upon the significant interactive effect of transect × season, pairwise PERMANOVA tests among the levels of the ‘season’ factor were performed separately for each transect (i.e., within each transect) as well as pairwise tests of the transects separately within each season. Each pair of seasons tested within each transect was significantly different (*p* ≤ 0.05; S3 Table) and the pairwise tests of the two transects within each season were all significantly different (*p* ≤ 0.05; S4 Table) as well.

**Table 3:** PERMANOVA results showing a statistically significant (*p* ≤ 0.05) difference in species composition among seasons and between transects.

	df	SS	MS	Pseudo-F	*p*	Permutations
**Transect**	1	8644.7	8644.7	5.4953	0.002	998
**Season**	3	2.80E+05	93285	59.3	0.001	998
**Transect x Season**	3	22121	7373.7	4.6873	0.001	999
**Residuals**	282	4.44E+05	1573.1			
**Total**	289	7.60E+05				

The top six species that contributed to the difference between transects had higher average abundances at the Cape Lookout transect, with the exception of Atlantic sharpnose, which had a higher average abundance at the Masonboro Inlet transect (S1 Table). The SIMPER analysis revealed that seasonal differences were largely attributable to spiny dogfish, clearnose skate, smooth dogfish (*Mustelus canis*), and smooth butterfly ray (S2 Table). For instance, spiny dogfish showed the highest average abundance in winter and none were caught in summer (Fig 4), and therefore spiny dogfish contributed the most to the differences in species composition in the winter when compared to any other season. Meanwhile, clearnose skate, smooth dogfish, and smooth butterfly ray showed the highest average abundances in the spring. Smooth dogfish and clearnose skate were nearly completely absent in the summer season whereas smooth butterfly rays had the lowest average abundance in winter (S2 Table).

**Fig 5:**
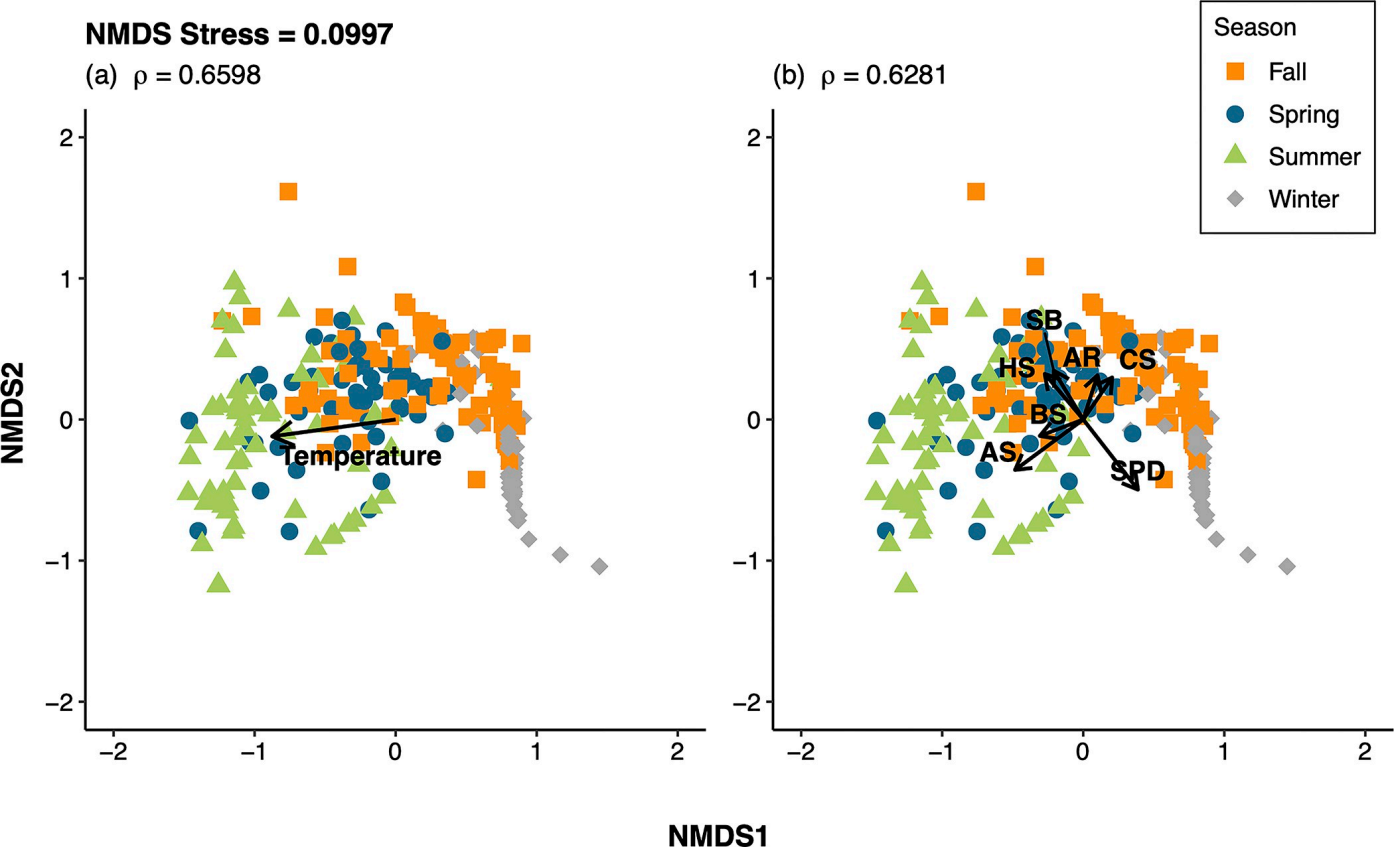
Non-metric multidimensional scaling (nMDS) ordinations of trawl tows among four seasons. Plot (a) displays the vector and Spearman correlation coefficient (*ρ* = 0.6495; Table 4) for temperature. Plot (b) displays the best-fitting combination of elasmobranch species driving seasonal differences and the species’ corresponding vectors. Species abbreviations are as follows: AS = Atlantic sharpnose; AR = Atlantic stingray; BS = blacknose shark; CS = clearnose skate; HS *= Hypanus spp*.; SB = smooth butterfly ray; SPD = spiny dogfish.

**Fig 6:**
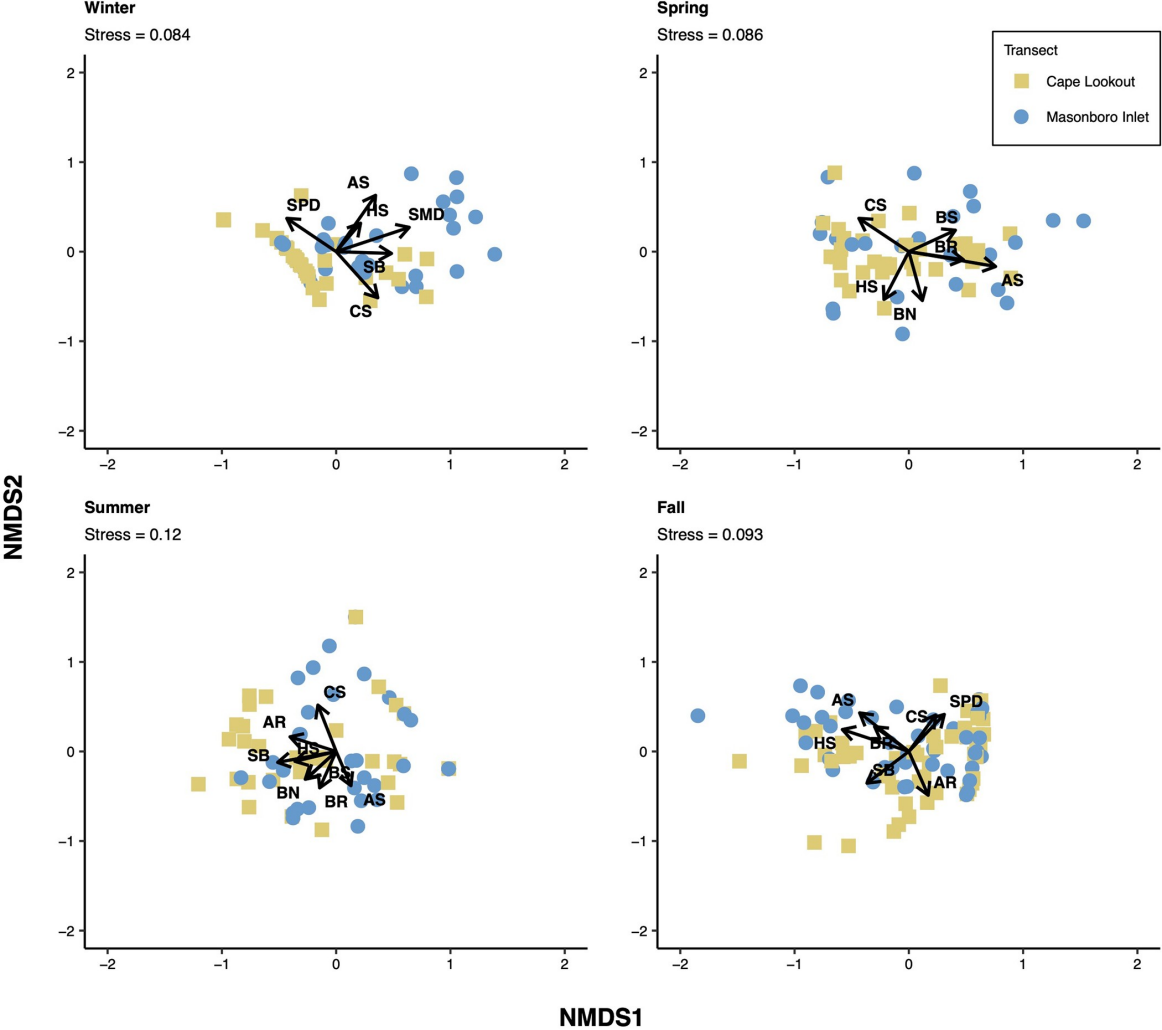
Non-metric multidimensional scaling (nMDS) ordinations of trawl tows from each season, indicating the transects by season. Each elasmobranch species in the combination that produced the greatest rank-correlation with sample dissimilarities of the given season are plotted as vectors. Species abbreviations are as follows: AS = Atlantic sharpnose; AR = Atlantic stingray; BS = blacknose shark; BN = bonnethead; BR = bullnose ray; CS = clearnose skate; HS = *Hypanus spp*.; SB = smooth butterfly ray; SMD = smooth dogfish; SPD = spiny dogfish.

Seasonal differences in community structure were apparent based on the nMDS ordinations, with winter and summer samples being the most distant in ordination space, while spring and fall assemblages were intermediate (Fig 5A). Winter showed the least amount of overlap with other seasons. With samples from the two transects combined, temperature produced the greatest Spearman rank correlation with the community composition, while the combination of all three environmental variables (temperature, salinity, and distance from shore-location) produced the lowest rank correlation (Table 4, Fig 5A). Temperature was the only variable included in the most parsimonious BIO-ENV model (Table 4) and was plotted as a vector on the nMDS ordination (Fig 5A). The spring and summer samples were characterized by warmer temperatures whereas the fall and winter samples were subject to lower temperatures, as expected (Fig 5A). Among the possible combinations of the ten species (i.e., species that comprised ≥1% of the total elasmobranch catch in any season), a combination of four species was most correlated with sample dissimilarities of the combined transects: Atlantic sharpnose, clearnose skate, smooth butterfly ray, and spiny dogfish (*ρ* = 0.6281; Table 5, Fig 5B). Overall correlation values were relatively high (0.4397–0.6281; Table 5). The spring, summer, and fall samples were characterized by several main species, but spiny dogfish largely drove the winter assemblage (Fig 5B). When considering samples for each transect separately, smooth dogfish appeared to contribute to differences between the two transects in the winter more than spiny dogfish (Fig 6). For the remaining three seasons, the differences in species assemblages driving sample dissimilarities at each transect were less distinct (Fig 6).

**Table 4:** BIO-ENV results showing the best-fitting groups of environmental variables (greatest Spearman correlations) driving the differences in the overall elasmobranch community among all seasons, at each transect and with transects pooled.

Cape Lookout transect				
**# of Variables**	** **	**Spearman Correlation (ρ)**	** **	**Variables Selected**
1		0.6132		Temperature
2		0.4236		Temperature, salinity
3		0.3225		Temperature, salinity, location
Masonboro Inlet transect				
**# of Variables**	** **	**Spearman Correlation (ρ)**	** **	**Variables Selected**
1		0.7355		Temperature
2		0.5486		Temperature, salinity
3		0.4528		Temperature, salinity, location
Transects pooled				
**# of Variables**	** **	**Spearman Correlation (ρ)**	** **	**Variables Selected**
1		0.6598		Temperature
2		0.4662		Temperature, salinity
3		0.4662		Temperature, salinity, location

**Table 5:** BIO-ENV results showing the best-fitting groups of species (greatest Spearman correlations) driving the differences in the overall elasmobranch community among all seasons.

# of Variables	Spearman Correlation (ρ)	Variables Selected
4	0.6281	Atlantic sharpnose, clearnose skate, smooth butterfly ray, spiny dogfish
3	0.626	Atlantic sharpnose, clearnose skate, spiny dogfish
5	0.6138	Atlantic sharpnose, blacknose shark, clearnose skate, smooth butterfly ray, spiny dogfish
6	0.5981	Atlantic sharpnose, blacknose shark, clearnose skate, *Hypanus spp*., smooth butterfly ray, spiny dogfish
7	0.5661	Atlantic sharpnose, blacknose shark, clearnose skate, *Hypanus spp*., smooth butterfly ray, smooth dogfish, spiny dogfish
2	0.5532	Atlantic sharpnose, clearnose skate
8	0.5369	Atlantic sharpnose, blacknose shark, bonnethead shark, clearnose skate, *Hypanus spp*., smooth butterfly ray, smooth dogfish, spiny dogfish
9	0.5125	Atlantic sharpnose, Atlantic stingray, blacknose shark, bonnethead shark, clearnose skate, *Hypanus spp*., smooth butterfly ray, smooth dogfish, spiny dogfish
10	0.4832	Atlantic sharpnose, Atlantic stingray, blacknose shark, bonnethead shark, bullose ray, clearnose skate, *Hypanus spp*., smooth butterfly ray, smooth dogfish, spiny dogfish
1	0.4397	Atlantic sharpnose

## Discussion

While the sampling was only truly year-round in 2005 and 2006, our sampling of the elasmobranch community on the inner continental shelf of North Carolina during every season over several years revealed clear seasonal and regional patterns in the species assemblage, reflecting the way each species uses these ocean habitats off North Carolina. These seasonal differences are expected to result from the latitudinal and depth-based seasonal migrations that these mobile species undertake. We also found that for some species, one sex utilized the surveyed habitats more than the other, and that differences in length distributions between males and females were common among the dominant species.

Overall, this study provides a more comprehensive analysis of both the shark and ray assemblages in the coastal waters of North Carolina than has been previously documented from other surveys [3,72]. It is important to understand elasmobranch community dynamics in the context of trophic interactions and the role these species play in the ecosystem [21], considering that elasmobranchs serve as both predators and prey in the nearshore shelf community [57,73,74]. For example, Woodland et al. [74] found that mesopredatory elasmobranchs such as smooth dogfish and clearnose skate showed niche overlap with teleost species, which could have implications for competition of resources. Bangley and Rulifson [39] estimated that the consumption of Atlantic menhaden (*Brevoortia tyrannus*) by overwintering mature spiny dogfish comprised the majority of the annual predatory impact on menhaden in North Carolina waters. Additionally, batoids in coastal nursery areas hold several key ecological roles as mesopredators, energetic links between habitats, and bioturbators in soft sediments [75].

The diverse batoid community in the coastal waters of Onslow Bay is evidenced herein and presents the possibility for future investigations to gather much-needed data on this group of species. Skates and rays comprise the majority of chondrichthyan species (51.8%) [10] yet information is often lacking on ecology, biology, and life history, and many batoid species are assessed as “data deficient” by the IUCN Red List [76,77]. Catches of skates and rays are unregulated in many regions of the U.S., including North Carolina [78–80], yet are frequently caught as trawl bycatch and are often recorded under an aggregated (e.g., “skates and rays”) category [21], which can obscure changes in the abundances of particular species and the overall community structure [57]. Certain batoid species such as the smooth butterfly ray, bullnose ray, and clearnose skate were relatively abundant in our dataset and are commonly observed in coastal surveys in the region [3,81], yet contemporary studies regarding the ecology of these species off the southeast U.S. are limited [57,82] and there is still a paucity of information on the seasonal distribution and movement patterns across the full continental shelf in this region. The presence of only juvenile clearnose skate in this survey may warrant future investigation into the coastal waters of Onslow Bay as a potential nursery area; however, further criteria would need to be investigated, such as comparisons of area densities and use patterns [75].

### Seasonal differences in community structure

Temperature was correlated with the seasonal dissimilarities in the species assemblages. Temperature is a well-known driver of elasmobranch movement and habitat use and is often a migration cue [83]. Ulrich et al. [6] also found that the seasonality of sharks in the nearshore and estuarine waters of South Carolina were influenced by temperature, with certain species (e.g., spiny dogfish, smooth dogfish) captured when water temperatures decreased below a certain threshold, followed by emigration from the area when temperatures returned to warmer levels. Indeed, during seasons when temperatures were changing rapidly, we would observe markedly different elasmobranch communities from one month to the next.

The clear seasonality of certain species, especially those that are highly migratory in the north-south direction (e.g., spiny dogfish) [84], lends credence to the argument that differences in species composition among seasons is likely linked to temperature differences. This dataset shows how the Onslow Bay elasmobranch community changes throughout the year, due to species that overwinter in the area as well as those that might remain in the Onslow Bay but migrate further inshore or offshore depending on the season (e.g., Atlantic sharpnose). Nonetheless, it is evident that the inner continental shelf is important for most elasmobranch species in North Carolina for at least part of the year, and these taxa are an important part of the nearshore shelf faunal community.

The correlation of temperature to the elasmobranch assemblages in this study increases the likelihood of ocean warming having major impacts on this group of species. Indeed, winter temperatures off the southeast U.S. coast have been shown to have a predictable influence on the annual community structure of marine coastal fauna based on species temperature preferences [85]. Future changes in the elasmobranch assemblage could occur as a result of shifting phenology, which will affect the timing of when different species occupy coastal habitats in North Carolina, and some species may experience major changes in abundance. Considering Onslow Bay’s location near the biogeographic transition zone at Cape Hatteras, we expect that the occurrence of more temperate species that seasonally range south of that break will diminish. Climate change may shift communities in warm temperate waters, like the southeast U.S., towards more subtropical and tropical species [34,85–87]. Spiny dogfish and clearnose skate have both been assessed to have a high potential for changes in geographic distribution, which is further supported by future projections of thermal habitat for these species [85,88]. However, changes in a community as a whole due to ocean warming can be highly uncertain due to the varying responses of the constituent species and the trophic interactions between them. Therefore, a better understanding of the population dynamics and species interactions of this assemblage will enhance our ability to engage in climate adaptive fisheries management [89].

### Spatial differences in community structure

Species composition differed between the Cape Lookout and Masonboro Inlet transects, possibly attributed to differences in regional bottom type, distance to the Gulf Stream, and proximity to a coastal feature (i.e., Cape Lookout) between these two transects. The Cape Lookout area is around 90 km closer to the Gulf Stream than the Masonboro Inlet area, which could explain the greater abundances and diversity of elasmobranch species caught at the Cape Lookout transect as the Gulf Stream’s warm waters support diverse fauna [90] and high productivity [91]. Numerous shark species have been shown to utilize the Gulf Stream area [92–94], likely due to the relative abundance of prey in the highly productive waters [92].

The biogeographic break between temperate and subtropical waters at Cape Hatteras could offer another possibility for differences in species composition between the two transects. The Cape Lookout transect is roughly 120 km closer to Cape Hatteras, and the species caught at the Cape Lookout transect but not the Masonboro Inlet transect include the sandbar shark (*Carcharhinus plumbeus)*, sand tiger shark (*Carcharias taurus*), Atlantic angel shark *(Squatina dumeril*), and the common thresher shark (*Alopias vulpius*). While all these species are known to inhabit both temperate and subtropical waters [95–99], the waters near Cape Hatteras and/or Cape Lookout have been shown to be of greater habitat importance for certain species [98]. However, these species were relatively uncommon in the catch (*n* < 10) and there may have been other factors contributing to the differences in the transects. Future studies comparing the community composition of catches closer to or at Cape Hatteras to those of the Onslow Bay transects would help elucidate how the biogeographic break influences species composition.

While multiple studies of elasmobranch communities document other major abiotic factors influencing the community dynamics, such as salinity or water clarity [100,101], these studies often focus on estuarine systems with greater mixing of tidally-influenced fresh, brackish, and saltwater leading to greater spatial differences in habitat features. Here, it was not surprising that salinity alone did not drive differences in the elasmobranch community, as the salinity range in nearshore waters fluctuates less than in estuarine systems. Further, our use of a bottom trawl limited our sampling to soft-bottom habitats in Onslow Bay. It remains unknown if the elasmobranch community on more complex seafloor habitats (e.g., live-bottom habitat on exposed rock shelves and ledges) in this region is distinct from the community that we sampled.

### Gear biases

All studies that examine structure of marine communities are subject to bias associated with gear selectivity. Our use of a trawl net in this survey may have yielded a bias towards benthic-oriented species and may have reduced our ability to quantify the importance of certain species utilizing higher portions of the water column (e.g., cownose ray). Additionally, the results may be biased toward smaller elasmobranch species that are less able to outswim the trawl. Highly mobile species likely residing in Onslow Bay may not be adequately represented here (e.g., sandbar shark) due to these species being able to outswim the net [102]. Longline sampling gear are commonly used to survey shark populations but may be biased towards more pelagic-oriented (i.e., inhabit the water column) or more benthic-oriented species depending on where the bait is positioned in the water column [103], and bias in estimated length of the community can occur due to hook size [104]. Indeed, Benavides [22] found a higher percentage of pelagic-oriented sharks caught in a multi-decadal longline shark survey in Onslow Bay, NC.

The type of trawl used in our study proved to be effective at sampling a large portion of the elasmobranch community, perhaps with the exception of highly mobile species. The net was chosen based on a wide vertical opening (~3.8 m) in order to effectively sample juvenile bluefish, which is a pelagic species [36]. Benthic species were also effectively sampled, based on the high catch rates of skates and benthic rays. Below, we highlight the patterns in seasonal occurrence, length distribution, and sex ratio for three of the commonly caught species in order to describe the most common uses of this nearshore habitat by this assemblage.

### Spiny dogfish

Spiny dogfish are known to overwinter in North Carolina and Virginia waters, [37,39,84,105]. Our results support that North Carolina, and specifically Onslow Bay, serves as important overwintering habitat for this migratory species, consistent with previous studies’ findings [37,39,84,105]. Spiny dogfish dominated the abundance of elasmobranchs in winter, more so than any other species during other seasons. The predatory impact of this temperate species on the overwintering fishes off the southeast U.S. might be large, considering the species’ opportunistic feeding and predation on the important forage fish Atlantic menhaden *(Brevoortia tyrannus)* in the southeastern U.S. [39].

The majority of the spiny dogfish catch in the nearshore waters of Onslow Bay was dominated by mature females (Fig 2). Past studies have shown that females in the northwest Atlantic are generally larger than males, occupy shallower waters than males [106–109], and larger, mature individuals often inhabit shallower waters closer inshore than their immature conspecifics [108–112]. Future research should examine the reproductive stages of mature females to determine how the overwintering time period relates to pupping. In our study, we found no evidence that pupping takes place off North Carolina, but aborted fetal sharks with egg yolk still attached were occasionally recovered from the catch.

### Clearnose skate

Clearnose skate was the only species to be one of the most abundant during all seasons. We found the lowest catches for this species during summer (Fig 4), which was similar to Schwartz [58] who did not report captures of clearnose skates between June and October in higher water temperatures. In the mid-Atlantic this species occupies estuarine water during summer [57]. However, in North Carolina they probably move offshore or northward into cooler water during the summer, because they are rarely caught during the summer and fall trawl survey conducted in Pamlico Sound, North Carolina [113]. Considering the high abundance of this species throughout the year, clearnose skate is probably an important benthic predator of invertebrates and fish in coastal systems [57]. Clearnose skates were largely caught as juveniles in this study (Fig 2), aligning with similar results found in trawl catches near Cape Lookout by Schwartz [58], indicating that the nearshore waters of Onslow Bay are important habitat for this life stage of the species.

While this study provides important fishery-independent data on clearnose skate distribution within nearshore waters of North Carolina, further research on the life history and habitat use of this species is still needed. In the northeastern U.S., clearnose skate is one of seven skate species managed as a single complex by the New England Fishery Management Council from the Gulf of Maine to Cape Hatteras [114] but is not federally managed in the south Atlantic. Landings of skate species are often reported in aggregate, as “unclassified skates” or “skates spp.” for fisheries management [57]. However, each species in the skate complex has a unique thermal and geographic range [114]. Previous to our study, data have been lacking for clearnose skate south of Cape Hatteras [114], but here we show that clearnose skate are found in waters between 9.2 to 28.6°C south of Cape Hatteras mainly in the months of October to June.

### Atlantic sharpnose

Use of the nearshore habitats of North Carolina by Atlantic sharpnose was almost entirely restricted to the summer season, when they were one of the most abundant species, consistent with previous studies [6,40,42,115]. Indeed, the complete absence of Atlantic sharpnose at cooler temperatures compared to their high abundance in summer trawls (Fig 4) led the species to be retained in every combination of the best-fit group of species driving the overall elasmobranch community among seasons. Seasonal differences in abundance in nearshore waters of North Carolina are probably driven by both north-south [116] and onshore-offshore movements; the authors have observed adults of this species occupying offshore wrecks in North Carolina during the winter.

Two distinct cohorts of Atlantic sharpnose were caught in this study—a more abundant group of immature individuals and another group of mature sharks. Most of the immature Atlantic sharpnose may have been young-of-the-year, suggesting nearby pupping locations, which is supported by other studies conducted off the southeast U.S. coast [6,40,42]. While the condition or healing stage of umbilical scars (an indicator of estimated age) [40] was not recorded here, Atlantic sharpnose of the size range known to be neonates with umbilical scars [40] were caught in our study, with a minimum fork length of 188 mm and 270 individuals less than 350 mm FL. This indicates that neonates were likely born in the shallow nearshore waters of Onslow Bay in the summer months and utilized the area in their first few months of life before migrating out of nearshore habitats. Future studies should investigate if the coastal waters in Onslow Bay fulfill the criteria of Heupel et al. [117] to be considered primary nursery grounds for Atlantic sharpnose.

## Conclusions

Understanding community dynamics, trophic interactions, and essential habitats across life stages are all essential factors to consider for ecosystem-based fisheries management (EBFM) [118]. It is also important to assess how human uses might affect coastal ecosystems, such as through fisheries interactions with target or non-target species. Sharks are targeted by both recreational and commercial fisheries on the Atlantic U.S. coast, with rod and reel and gillnet, respectively [119,120]. As such, research priorities for managed species include identification and characterization of key habitats [121,122], life history studies, and additional length and age sampling [111]. Stratton et al. [123] found that annual abundances of certain elasmobranch and fish species in the southeastern U.S. were negatively correlated with effort within the regional shrimp trawl fishery. Species sensitive to the trawling effort included many of the elasmobranchs caught in this study, including Atlantic stingray, bluntnose stingray, smooth butterfly ray, smooth dogfish, clearnose skate, Atlantic sharpnose, and bonnethead. For such species, population responses to fisheries interactions should be monitored closely. Additionally, other ecosystem uses, such as offshore energy development and marine sediment mining, may impact essential habitats. It is important to understand the habitat use of these species and how mitigation of anthropogenic activities such as seasonal timing for mining or dredging may be able to lessen disruptions to migrations or seasonal presence of important species [37]. Link [118] stressed that monitoring programs should include habitat characterization, environmental variables, food habits, and non-target species in order to successfully approach fisheries management from an ecosystem view. This study has presented an overview of the elasmobranch community found in the shallow nearshore waters of North Carolina; the species’ patterns of seasonal occurrence can help to inform the broader view of the marine ecosystem in the area and also help in the understanding of recent [124] potential climate change impacts on the coastal assemblage.

## Supporting information

S1 Table SIMPER results showing species contributions to statistically significant (PERMANOVA, p ≤ 0.05) differences in species composition between transects (CL = Cape Lookout, MI = Masonboro Inlet) for all seasons combined. (TIFF)

S2 Table SIMPER results showing species contributions to statistically significant (PERMANOVA, *p* ≤ 0.05) differences in species composition among seasons for both transects combined. (TIFF)

S3 Table PERMANOVA results showing statistically significant (*p* ≤ 0.05) differences in species composition among each pair of seasons tested within each transect and the two transects tested within each season. (TIFF)

S4 Table PERMANOVA results showing statistically significant (*p* ≤ 0.05) differences in species composition among the two transects tested within each season. (TIFF)
