# Supplementary figures and images for: Seasonality and relative abundance within an elasmobranch assemblage near a major biogeographic divide
Source: PLoS One. 2024 Jun 26;19(6):e0300697. doi: 10.1371/journal.pone.0300697 (PMC11207119; doi:10.1371/journal.pone.0300697)

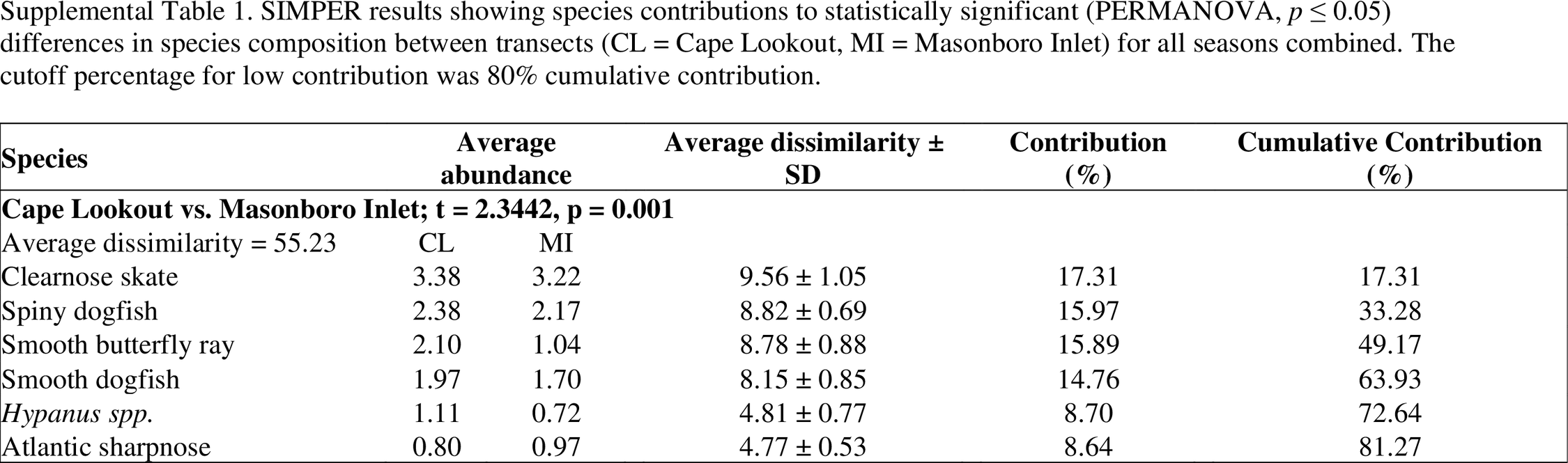

Supplement: S1 Table — (TIFF) [file pone.0300697.s001.tiff]

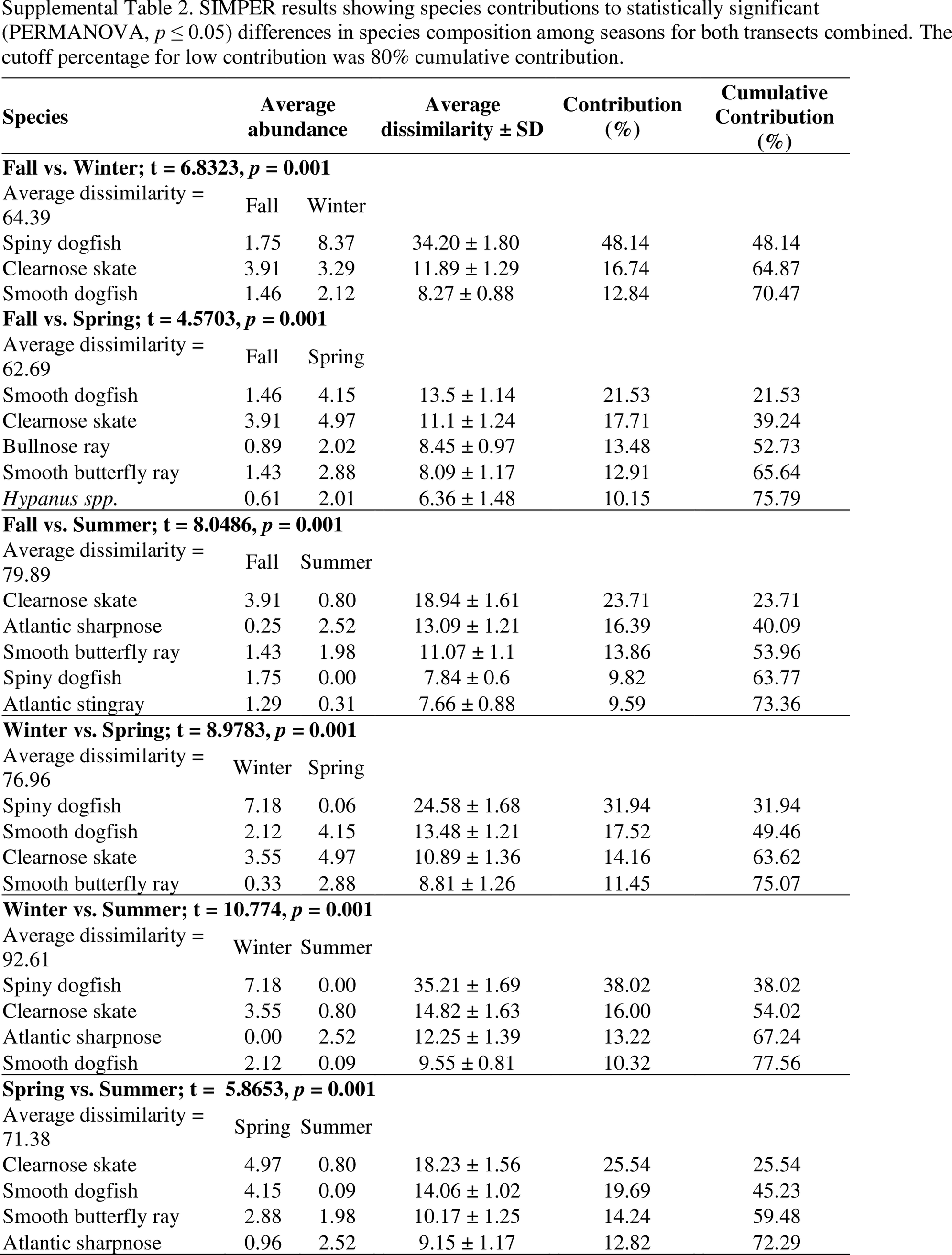

Supplement: S2 Table — (TIFF) [file pone.0300697.s002.tiff]

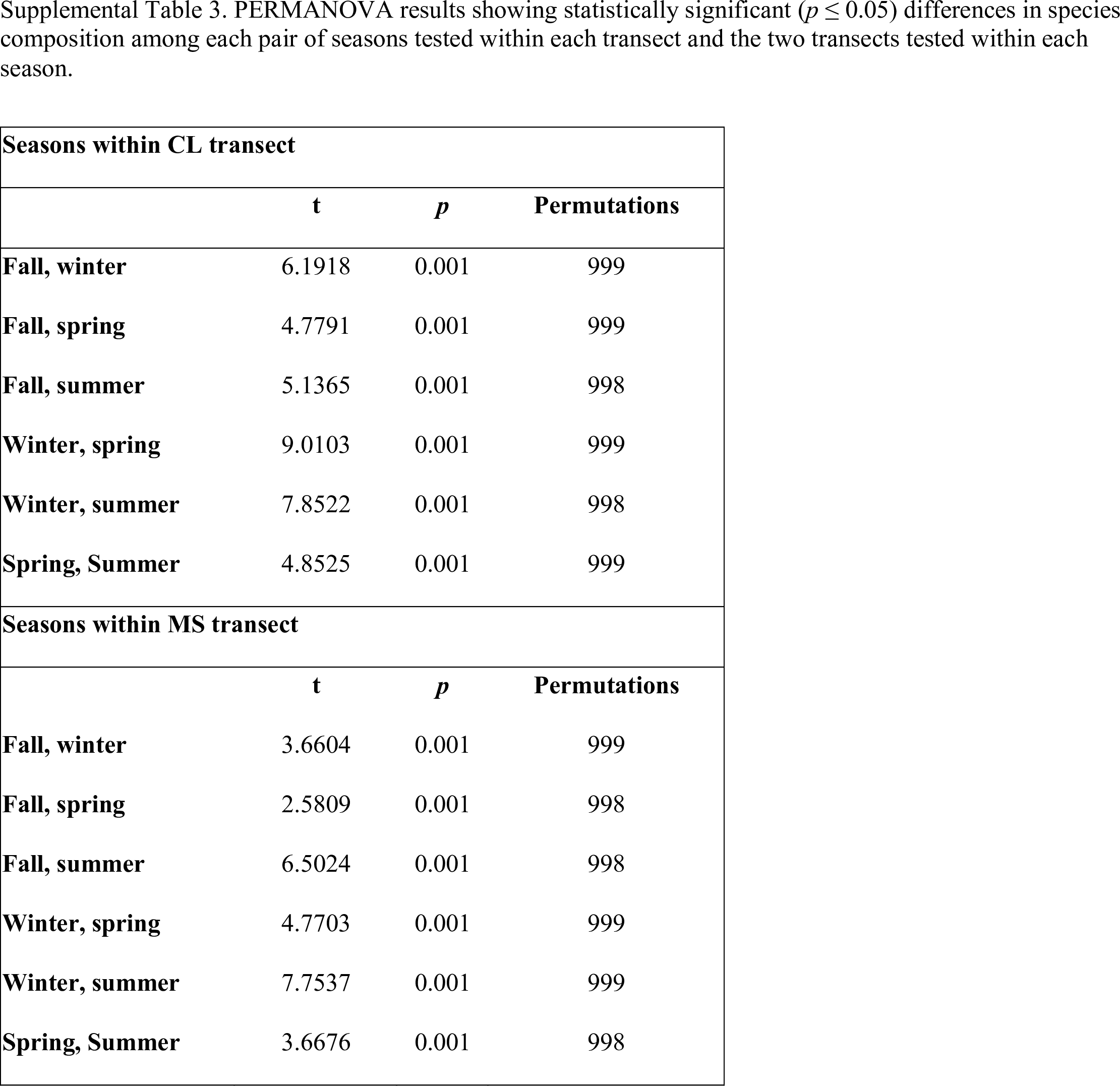

Supplement: S3 Table — (TIFF) [file pone.0300697.s003.tiff]

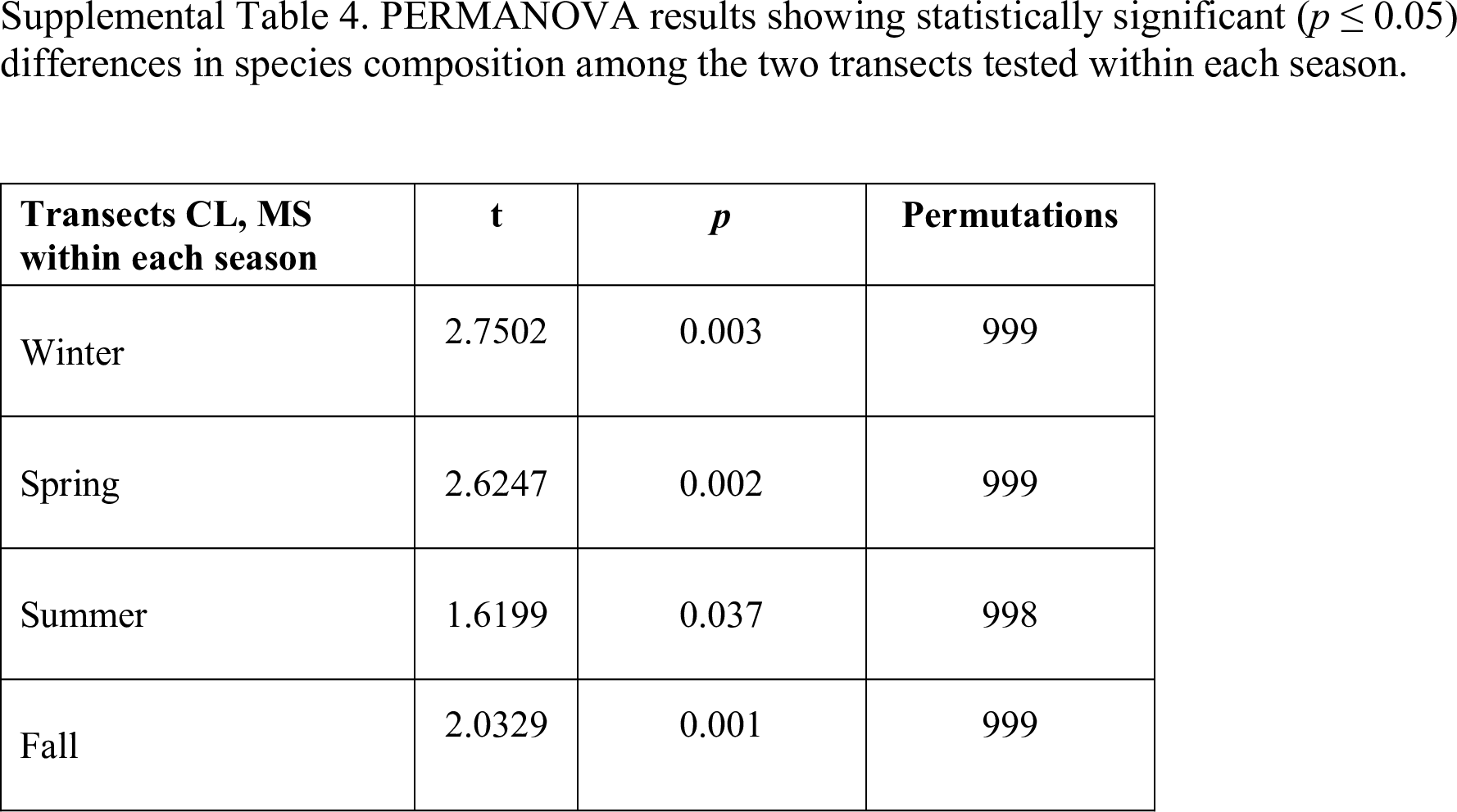

Supplement: S4 Table — (TIFF) [file pone.0300697.s004.tiff]
